# ChREBP regulates *Pdx-1* and other glucose-sensitive genes in pancreatic β-cells

**DOI:** 10.1016/j.bbrc.2010.10.010

**Published:** 2010-11-12

**Authors:** Gabriela da Silva Xavier, Gao Sun, Qingwen Qian, Guy A. Rutter, Isabelle Leclerc

**Affiliations:** Section of Cell Biology, Division of Diabetes, Endocrinology & Metabolism, Department of Medicine, Faculty of Medicine, Imperial College London, UK

**Keywords:** ARNT, aryl hydrocarbon receptor nuclear translocator, ChREBP, carbohydrate responsive element-binding protein, ChIP, chromatin immunoprecipitation, ChoRE, carbohydrate-responsive element, FAS, fatty acid synthase, GcK, glucokinase, GFP, green fluorescent protein, HIF, hypoxia inducible factor, L-PK, L-type pyruvate kinase, Pdx-1, pancreatic and duodenum homeobox-1, siRNA, small interfering RNA, SREBP-1c, sterol regulatory response-element-binding protein-1c, USF, upstream stimulatory factor, ChREBP, Pdx-1, MafA, Insulin, Glucokinase, Gene expression, Pancreatic β-cells, MIN6, Islets of Langerhans

## Abstract

Carbohydrate responsive element-binding protein (ChREBP) is a transcription factor whose expression and activity are increased in pancreatic β-cells maintained at elevated glucose concentrations. We show here that ChREBP inactivation in clonal pancreatic MIN6 β-cells results in an increase in *Pdx-1* expression at low glucose and to a small, but significant, increase in *Ins2*, *GcK* and *MafA* gene expression at high glucose concentrations. Conversely, adenovirus-mediated over-expression of ChREBP in mouse pancreatic islets results in decreases in Pdx-1, MafA, Ins1, Ins2 and GcK mRNA levels. These data suggest that strategies to reduce ChREBP activity might protect against β-cell dysfunction in type 2 diabetes.

## Research highlights

► ChREBP silencing enhances glucose-responsive gene expression in MIN6 β-cells. ► ChREBP modulation of *Pdx-1* gene expression might be indirect. ► ChREBP over-expression decreases Pdx-1, MafA, Ins1, Ins2 and GcK mRNA levels in mouse pancreatic islets of Langerhans.

## 1 Introduction

Pancreatic β-cell glucolipotoxicity [1] is considered to play a significant role in the pathogenesis of type 2 diabetes. Carbohydrate responsive element-binding protein (ChREBP) is a member of the basic helix–loop–helix family of transcription factors and transactivates glucose-responsive genes by binding to DNA as a heterodimer with Max-like protein X1 at a well-defined carbohydrate-responsive element (ChoRE) [2–5]. In the liver, ChREBP is responsible for converting excess carbohydrate to fatty acids for long-term storage [6]. Mice deleted for both alleles of ChREBP display diminished rates of hepatic glycolysis and lipogenesis resulting in high liver glycogen content, low plasma free fatty acid levels and reduced adipose tissue mass [7]. Loss of ChREBP in leptin-null *ob*/*ob* mice protects against obesity [7,8].

We, and others, have previously shown that, in pancreatic β-cells, ChREBP is activated by high glucose and is responsible for the induction of the lipogenic genes fatty acid synthase (FAS) and L-type pyruvate kinase (L-PK) [9,10], and the proapoptotic gene *Txnip* [11,12]. ChREBP also represses aryl hydrocarbon receptor nuclear translocator/hypoxia inducible factor 1-β (ARNT/HIF1-β) [13] shown recently to be diminished in islets [14] and liver [15] of type 2 diabetic humans, and necessary for normal β-cell function and repression of hepatic gluconeogenesis. We sought here to investigate the effects of ChREBP silencing and over-expression on other key glucose-responsive genes in pancreatic islet β-cells, namely pancreatic and duodenal homeobox-1 (Pdx-1), MafA, glucokinase (GcK) and insulin, all critical for normal pancreatic β-cell function.

## 2 Materials and methods

### 2.1 Materials

Primers for siRNA construction and PCR were from MWG Biotech (Milton Keynes, UK). Antibodies were described in [9]. Other reagents were from Sigma or Invitrogen.

### 2.2 Plasmids, adenoviruses and siRNA

pChREBP and ChREBP siRNA have been described in [9]. Adenovirus encoding for ChREBP has been described in [13]. Plasmids and adenoviruses encoding GFP-null and constitutively active and dominant negative forms of SREBP-1c were described in [16]. pPdx1.Luc_FF_, encoding the 5′ flanking region of the mouse pancreatic duodenum homeobox-1 (*Pdx-1*) gene (−2715 to 0 bp), was generated by PCR from MIN6 cell genomic DNA with the following primers: forward, 5′-ATAT GG TACC CTC CAG TAT CAG GGA GGA-3′ (KpnI site underlined); reverse, 5′-TTT GAGCTC CCA CCC CAG ATC GCT TTG A-3′ (SacI site underlined) and subcloned into pGL3 basic (Promega). Two point mutations [−106C > A; −102T > G] in the *Pdx-1* promoter were introduced using Quickchange™ (Stratagene) with the following sense primer: 5′-ATG GCT CCA GGG TAA ACA ACG GGG GGT GCC CCA GAG CCT ATG-3′.

### 2.3 MIN6 cell culture and islet of Langerhans isolation

MIN6 cells were cultured as in [9]. Mouse islets of Langerhans were isolated and cultured as in [13].

### 2.4 Single cell reporter gene assay

Intranuclear microinjection of plasmids, antibodies and siRNAs in MIN6 cells were performed at plasmid concentrations of 0.1 (pPdx1.Luc_FF_), and 0.05 (pChREBP, pSREBP-1c, pCMV-RL) mg ml^−1^, and antibody against ChREBP and SREBP at 1 mg ml^−1^, before imaging as described in [9].

### 2.5 Real-time RT-PCR

Total mRNA isolation, cDNA generation and real-time quantitative PCR were performed with primers listed in Table 1, as in [13] and according to the manufacturer’s instructions. Levels of mRNA encoding the indicated genes were normalized compared with cyclophilin mRNA and expressed as the fold change over control (null, 3 mM glucose) and presented as the means ± SEM.

### 2.6 Chromatin immunoprecipitation assay

Chromatin immunoprecipitation was performed essentially as described in [9,13].

### 2.7 Statistical analysis

Data are given as means ± SEM. Comparisons between means were performed by unpaired two-tailed Student’s *t*-test with Bonferroni correction as appropriate, using Microsoft Excel.

## 3 Results

### 3.1 ChREBP silencing enhances glucose-responsive gene expression in MIN6 pancreatic β-cells

**Fig. 1:**
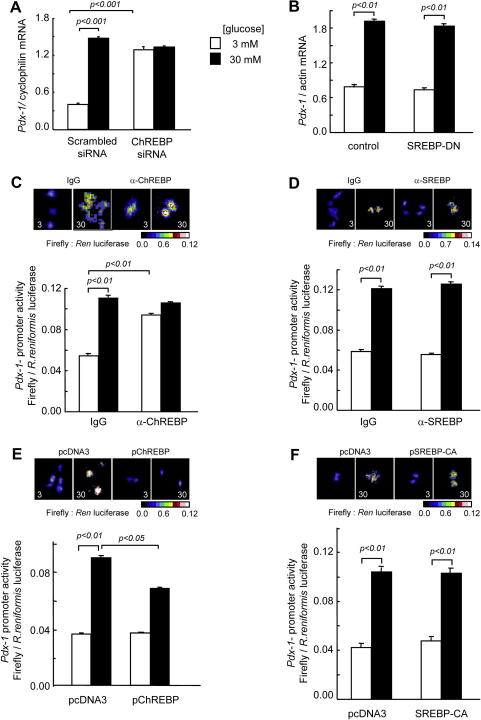
ChREBP is a repressor of *Pdx-1* gene expression in MIN6 cells. (A,B) MIN6 cells were cultured for 48 h in the presence of scrambled or ChREBP siRNA (A), or in the presence of null or SREBP-DN adenoviruses (B), then overnight in 3 mM glucose and finally for 6 h in 3 or 30 mM glucose prior to cell lysis, total RNA extraction and real-time quantitative RT-PCR (see Section 2). (C,D) *Pdx-1* promoter activity was monitored via nuclear and cytoplasmic microinjection of *Pdx-1* promoter–reporter system and anti-ChREBP (C) or anti-SREBP (D) antibodies (1 mg ml^−1^), or control IgG as indicated, before culture at the indicated glucose concentrations for 6 h and luciferase imaging as described in Section 2. (E,F) Pdx-1 promoter activity was monitored as above but after co-microinjection of pChREBP (E) or SREBP-CA (F) plasmids. Data are the means ± SEM of three separate experiments.

We have previously shown that ChREBP silencing in pancreatic murine insulinoma MIN6 β-cells improves glucose-stimulated insulin secretion, possibly through a decrease in total triglyceride content [9]. Here, we examined the impact of ChREBP silencing by RNA interference on other glucose-responsive genes in MIN6 β-cells. ChREBP knockdown increased the levels of mRNA encoding *MafA*, *GcK* and *Ins2* at high (30 mM) glucose concentrations, whereas ChREBP silencing increased the expression of the *Pdx-1* gene at low (3 mM) glucose concentrations (Table 2 and Fig. 1A). Correspondingly, we observed a similar increase in *Pdx-1* promoter activity at low glucose after ChREBP inhibition by microinjection of a specific anti-ChREBP antibody (Fig. 1C), while introduction of a ChREBP expression vector by microinjection suppressed the activity of *Pdx-1* promoter at high glucose (Fig. 1E). By contrast, SREBP1-c inactivation or over-expression was without effect on *Pdx-1* promoter activity or mRNA levels (Fig. 1B, D and F).

### 3.2 ChREBP modulation of *Pdx-1* gene expression might be indirect

**Fig. 2:**
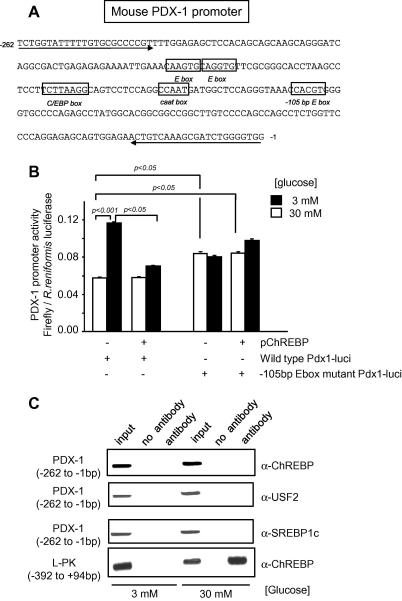
Chromatin immunoprecipitation fails to reveal direct ChREBP binding at the proximal Pdx-1 promoter. (A) DNA sequence of the proximal mouse Pdx-1 promoter showing relevant E-box motifs. Arrows represent the primers used for ChIP assay in (C). (B) Cells were microinjected with either wild-type or mutated pPdx-1.Luc_FF_ plus pCMV.RL, plus either empty pcDNA3 or pChREBP as indicated, and incubated and imaged as in Fig. 1. (C) MIN6 cells were cultured in medium containing 3 mM glucose for 16 h prior to stimulation with media containing 3 or 30 mM glucose for 24 h prior to chromatin immunoprecipitation using the antibodies and primers to amplify either Pdx-1 or L-PK promoter as indicated alongside the figure. Data are representative of three independent experiments.

**Fig. 3:**
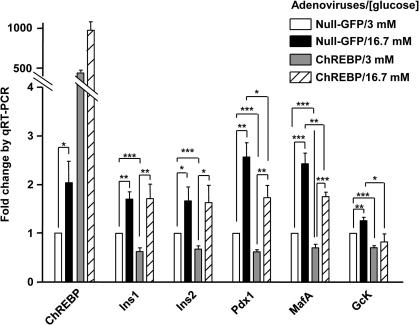
Sequences of the primers and E-boxes in the −2.7 kb Pdx-1 promoter probed by ChIP assay. Positions of 5′ and 3′ primers are indicated by arrows and those of E-boxes are highlighted in bold.

We next sought to identify the region on the *Pdx-1* promoter responsive to ChREBP repression. No consensus ChoRE exists on the *Pdx-1* promoter, but a proximal E-box, located at −105 bp (Fig. 2A) is highly conserved between species, is protected in DNAse footprints, and has been proposed to confer β-cell specificity to the *Pdx-1* promoter [17]. Up to now, it has been thought that this site predominantly binds USF, since mutations abolishing the binding of the latter factor impair the activity of the *Pdx-1* promoter, whereas over-expression of a dominant-negative USF2 reduces both *Pdx-1* promoter activity as well as Pdx-1 mRNA and protein levels [17,18]. Indeed, mutation of this site abolished both the glucose response and the repressive effect of ChREBP of the *Pdx-1* reporter construct (Fig. 2B). However, neither ChREBP, USF2 nor SREBP-1c binding could be detected to the proximal (−260 to +1) region of the promoter by chromatin immunoprecipitation (Fig. 2C). By contrast, and as previously reported [9], ChREBP binding was readily detectable on the proximal L-PK promoter at elevated glucose concentrations (Fig. 2C, bottom panel). We also used this approach to screen a further 11 E-boxes lying in the Pdx-1 promoter region between −2.7 and −0.26 kb (Fig. 3) but could not reveal evidence for ChREBP (not shown).

### 3.3 Adenovirus-mediated over-expression of ChREBP inhibits glucose-responsive gene expression in isolated mouse islets of Langerhans

**Fig. 4:**
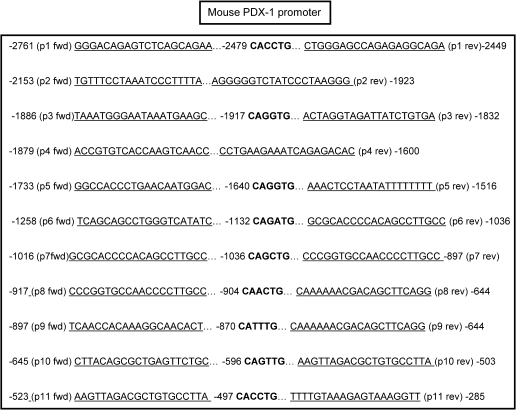
Real-time quantitative PCR analysis of gene expression in mouse islets over-expressing ChREBP. Islets of Langerhans isolated from female CD1 mice aged 12–14 weeks were transduced with either null-GFP or ChREBP adenoviruses (100 MOI) and cultured in 11 mM glucose RPMI media for 48 h and incubation in RPMI media supplemented with 3 or 16.7 mM glucose overnight prior to qRT-PCR as described in methods. Data are means ± SEM from three independent experiments done in triplicates, and normalized to cyclophilin mRNA levels prior to expression as fold change from null infected islets cultured at 3 mM glucose. ^*^p *<* 0.05; ^**^p < 0.01; ^***^p < 0.001.

We next examined the effects of ChREBP over-expression on the levels of mRNAs encoding the above glucose-responsive genes in intact islets of Langerhans. Mouse islets were transduced either with a null adenovirus encoding GFP only or with an adenovirus encoding full length wild-type ChREBP [13]. ChREBP over-expression resulted in a decrease in the levels of the mRNA encoding *Pdx-1*, *MafA* and *GcK* at both low and high glucose concentrations, and *Ins1* and *Ins2* at low glucose concentrations only (Fig. 4).

## 4 Discussion

### 4.1 Defining a role for ChREBP in glucose-induced pancreatic β-cell dysfunction and diabetes

ChREBP is emerging as an important transcription factor in the pathogenesis of obesity and diabetes and their complications. Indeed, ChREBP is now strongly implicated in the pathogenesis of fatty liver disease and insulin resistance [8,19] acting to induce lipogenic genes. Very recently, ChREBP was proposed to contribute to the development of diabetic nephropathy through the induction of HIF1-α in glomerular mesangial cells [20,21] In the pancreatic β-cell, ChREBP activation by high glucose results in increases in triglyceride content and a reduction in glucose-stimulated insulin secretion [9,13]. We show here that, in addition to the direct effects on L-PK, FAS, Txnip and ARNT promoters previously reported [9,10,12,13] ChREBP also inhibits the expression of several other key β-cell genes, namely *Pdx-1*, *MafA*, *GcK* and *insulin*.

### 4.2 Regulation of *Pdx-1* gene expression by ChREBP

We show firstly that ChREBP over-expression, both in MIN6 β-cells and pancreatic islets, inhibits *Pdx*-1 gene expression at high glucose concentrations, and that ChREBP inactivation, achieved either by RNA silencing or antibody microinjection into single cells, increases *Pdx-1* expression at low glucose concentrations. Although ChREBP has been shown to possess intrinsic repressive capabilities [13,22], we were unable to demonstrate direct binding of ChREBP *in vivo* despite scanning the obvious E-boxes, by extensive ChIP assays, at either low or high glucose concentrations (see Fig. 3). We have considered the possibility that an indirect effect of changes in intracellular lipid content could explain the effects of ChREBP on *Pdx-1* gene expression, but this would appear to be unlikely given the absence of effects of either inactivation of endogenous SREBP-1c or over-expression of the activated nuclear fragment of SREBP-1c on *Pdx-1* promoter activity or mRNA levels (Fig. 1B, D and F), despite well-documented effects of these manoeuvres on cellular triglyceride content [16,23,24]. One possible explanation for the apparent inhibitory effects of ChREBP on *Pdx-1* gene expression at low glucose concentrations might be that ChREBP normally serves to sequester another activator(s) of *Pdx-1* under these conditions. Translocation of ChREBP to the nucleus at high glucose concentrations, and its activation, either through covalent modification through phosphorylation/dephosphorylation reactions [25], or through an ill-defined mechanism involving the N-terminal LID domain of ChREBP [26,27], may then release the bound transcriptional activator, allowing the latter to bind to the *Pdx-1* promoter to stimulate transcription.

It is of note that we were unable here to demonstrate the binding of USF2 at the −105 E-box of the *Pdx-1* promoter. Our ChIP assay using both anti-USF and anti-SREBP antibodies has been validated in this cell type on the FAS promoter [9], making a false negative unlikely. The discrepancy between data obtained from *in vitro* studies and our ChIP assay demonstrates the significance of *in vivo* (live cell) approaches. Further work will be necessary to identify the key transcription factor binding to the β-cell specific regulatory sequences of the proximal *Pdx-1* promoter.

### 4.3 Regulation of the insulin genes by ChREBP

ChREBP silencing in MIN6 cells resulted in a small, but significant, increase in *Ins2* gene expression whereas, in primary islets, ChREBP over-expression only decreased the amount of *Ins1* and *Ins2* mRNAs at low glucose, but not at elevated glucose concentrations. One possible explanation may lie in the extremely long half-life of insulin mRNAs at high glucose concentrations [28], therefore making a decrease in its transcription rate unnoticeable within the time course of these experiments.

## 5 Conclusion

We conclude that ChREBP is a key regulator of adult β-cell phenotype, affecting the expression of critical genes. It therefore seems possible that increases in ChREBP expression, prompted by a diabetic milieu, may exacerbate β-cell dysfunction and accelerate β-cell failure in type 2 diabetes. It would be interesting to know whether a β-cell specific ChREBP knockout mouse would be protected against the development of β-cell failure during the course of some forms of diabetes.

## Figures and Tables

**Table 1:** Primers used for real-time RT-PCR.

mRNA	Forward primer 5′–3′	Reverse primer 5′–3′
MafA	CACCACGTGCGCTTGG	CAGAAAGAAGTCGGGTG
Pdx-1	TGGAGCTGGCAGTGATGTTGA	TCAGAGGCAGATCTGGCCAT
Ins1	GAAGCGTGGCATTGTGGAT	TGGGCCTTAGTTGCAGTAGTTCT
Ins2	AGCCCTAAGTGATCCGCTACAA	CATGTTGAAACAATAACCTGGAAGA
GcK	GCTTTTGAGACCCGTTTTGTG	GCCTTCGGTCCCCAGAGT
Cyclophilin	TATCTGCACTGCCAAGACTGA	CCACAATGCTCATGCCTTCTTTCA

**Table 2:** Effects of glucose and ChREBP silencing on mRNA levels in MIN6 cells.

[Glucose]	Scrambled siRNA		ChREBP siRNA	
	3 mM	30 mM	3 mM	30 mM
MafA	0.0294 ± 0.0002	0.0332 ± 0.0002^***^	0.0297 ± 0.0002	0.0395 ± 0.0002^*,¶^
GcK	0.397 ± 0.0002	0.927 ± 0.0006^***^	0.386 ± 0.0002	1.14 ± 0.0001^***,¶^
Ins2	3.52 ± 0.004	9.79 ± 0.004^***^	3.48 ± 0.0007^¶^	10.2 ± 0.004^***,¶^

Culture conditions, total RNA preparation and real-time RT-PCR conditions were as described in Fig. 3. Data are means ± SEM from three independent experiments performed in triplicates, and normalized to cyclophilin mRNA levels. ^*^, ^***^ indicate *p* < 0.05, 0.0001 for the effect of glucose and ^¶^ indicates *p* < 0.05, for the effect of ChREBP siRNA.
